# Heavy metals removal from aqueous environments by electrocoagulation process– a systematic review

**DOI:** 10.1186/s40201-015-0233-8

**Published:** 2015-10-26

**Authors:** Edris Bazrafshan, Leili Mohammadi, Alireza Ansari-Moghaddam, Amir Hossein Mahvi

**Affiliations:** Health Promotion Research Center, Zahedan University of Medical Sciences, Zahedan, Iran; Department of Environmental Health Engineering, School of Public Health, Tehran University of Medical Sciences, Tehran, Iran; Center for Solid Waste Research, Institute for Environmental Research, Tehran University of Medical Sciences, Tehran, Iran; National Institute of Health Research, Tehran University of Medical Sciences,, Tehran, Iran

**Keywords:** Electrocoagulation, Wastewater treatment, Heavy metals removal

## Abstract

Heavy metals pollution has become a more serious environmental problem in the last several decades as a result releasing toxic materials into the environment. Various techniques such as physical, chemical, biological, advanced oxidation and electrochemical processes were used for the treatment of domestic, industrial and agricultural effluents. The commonly used conventional biological treatments processes are not only time consuming but also need large operational area. Accordingly, it seems that these methods are not cost-effective for effluent containing toxic elements. Advanced oxidation techniques result in high treatment cost and are generally used to obtain high purity grade water. The chemical coagulation technique is slow and generates large amount of sludge. Electrocoagulation is an electrochemical technique with many applications. This process has recently attracted attention as a potential technique for treating industrial wastewater due to its versatility and environmental compatibility. This process has been applied for the treatment of many kinds of wastewater such as landfill leachate, restaurant, carwash, slaughterhouse, textile, laundry, tannery, petroleum refinery wastewater and for removal of bacteria, arsenic, fluoride, pesticides and heavy metals from aqueous environments. The objective of the present manuscript is to review the potential of electrocoagulation process for the treatment of domestic, industrial and agricultural effluents, especially removal of heavy metals from aqueous environments. About 100 published studies (1977–2016) are reviewed in this paper. It is evident from the literature survey articles that electrocoagulation are the most frequently studied for the treatment of heavy metal wastewater.

## Introduction

Environmental issues, mainly concerning chemical and biological water pollution, represent a key priority for civil society, public authorities and, especially, for the industrial sector. In fact, the use of water, both in urban and industrial contexts, implies its subsequent pollution: any activity, whether domestic, agricultural or industrial, produces effluents containing undesirable, and possibly toxic, pollutants. Thus, a constant effort to protect water resources is being made by the various governments, through the introduction of increasingly strict legislation covering pollutant release. In particular for liquid industrial effluents, recent restrictions impose appropriate treatments of wastewater before its release into the environment [1].

This high pollutant load poses complex and extremely varied problems, related to each particular situation. In addition, the release of organic and inorganic pollutants is not uniform (either in quality or in quantity), but always leads to the same result: toxicity for aquatic ecosystems which creates worries for the population [2].

Industrial wastewaters like electroplating or acid mine wastewaters contain various kinds of toxic substances such as cyanides, alkaline cleaning agents, degreasing solvents, oil, fat and metals [3]. Most of the metals such as copper, nickel, chromium, silver and zinc are harmful when they are discharged without treatment [3]. Heavy metals are elements having atomic weights between 63.5 and 200.6 and a specific gravity greater than 5 [4].

With the rapid development of industries such as metal plating facilities, mining operations, fertilizer industries, tanneries, batteries, paper industries and pesticides, etc., heavy metals wastewaters are directly or indirectly discharged into the environment increasingly, especially in developing countries. Unlike organic contaminants, heavy metals are not biodegradable and tend to accumulate in living organisms and many heavy metal ions are known to be toxic or carcinogenic. Toxic heavy metals of particular concern in treatment of industrial wastewaters include zinc, copper, nickel, mercury, cadmium, lead and chromium. Zinc is a trace element that is essential for human health. It is important for the physiological functions of living tissue and regulates many biochemical processes. However, too much zinc can cause eminent health problems, such as stomach cramps, skin irritations, vomiting, nausea and anemia [5]. Copper does essential work in animal metabolism. But the excessive ingestion of copper brings about serious toxicological concerns, such as vomiting, cramps, convulsions, or even death [6]. Nickel exceeding its critical level might bring about serious lung and kidney problems aside from gastrointestinal distress, pulmonary fibrosis and skin dermatitis [7]. And it is known that nickel is human carcinogen. Mercury is a neurotoxin that can cause damage to the central nervous system. High concentrations of mercury cause impairment of pulmonary and kidney function, chest pain and dyspnea [8]. The classic example of mercury poisoning is Minamata Bay. Cadmium has been classified by U.S. Environmental Protection Agency as a probable human carcinogen. Cadmium exposes human health to severe risks. Chronic exposure of cadmium results in kidney dysfunction and high levels of exposure will result in death. Lead can cause central nervous system damage. Lead can also damage the kidney, liver and reproductive system, basic cellular processes and brain functions. The toxic symptoms are anemia, insomnia, headache, dizziness, and irritability, weakness of muscles, hallucination and renal damages [9]. Chromium exits in the aquatic environment mainly in two states: Cr^3+^ and Cr^6+^. In general, Cr^6+^is more toxic than Cr^3+^. Cr^6+^ affects human physiology, accumulates in the food chain and causes severe health problems ranging from simple skin irritation to lung carcinoma [10]. Various regulatory bodies have set the maximum prescribed limits for the discharge of toxic heavy metals in the aquatic systems. However the metal ions are being added to the water stream at a much higher concentration than the prescribed limits by industrial activities, thus leading to the health hazards and environmental degradation (some of permissible limits and health effects of various toxic heavy metals are presented Table 1).

**Table 1:** Permissible limits and health effects of various toxic heavy metals

Metal contaminant	Permissible limits for industrial effluent discharge (in mg/l)						Permissible limits by international bodies (μg/l)		Health hazards
	Into inland surface waters Indian Standards: 2490 (1974)^a^	Into inland surface waters, Iranian Standards (2009)^b^	Into public sewers, Indian Standards: 3306 (1974)^a^	Into public sewers, Iranian Standards (2009)^b^	On land for irrigation, Indian Standards: 3307 (1974)^a^	On land for irrigation, Iranian Standards (2009)^b^	WHO ^a^	USEPA^a^	
Arsenic	0.20	0.10	0.20	0.10	0.20	0.10	10	50	Carcinogenic, producing liver tumors, skin and gastrointestinal effects
Mercury	0.01	-	0.01	-	–	-	01	02	Corrosive to skin, eyes and muscle membrane, dermatitis, anorexia, kidney damage and severe muscle pain
Cadmium	2.00	0.10	1.00	0.10	–	0.05	03	05	Carcinogenic, cause lung fibrosis, dyspnea and weight loss
Lead	0.10	1.00	1.00	1.00	–	1.00	10	05	Suspected carcinogen, loss of appetite, anemia, muscle and joint pains, diminishing IQ, cause sterility, kidney problem and high blood pressure
Chromium	0.10	Cr^6+^ = 0.50, Cr^3+^ = 2.0	2.00	Cr^6+^ = −, Cr^3+^ = 2.0	–	Cr^6+^ = 1.00, Cr^3+^ = 2.0	50	100	Suspected human Carcinogen, producing lung tumors, allergic dermatitis
Nickel	3.0	2.0	3.0	2.0	–	2.0	–	–	Causes chronic bronchitis, reduced lung function, cancer of lungs and nasal sinus
Zinc	5.00	2.0	15.00	2.0	–	2.0	–	–	Causes short-term illness called “metal fume fever” and restlessness
Copper	3.00	1.0	3.00	1.0	–	0.2	–	1300	Long term exposure causes irritation of nose, mouth, eyes, headache, stomachache, dizziness, diarrhea

In the above Table ^a^referred to Reference No. [61] and ^b^referred to Reference No. [62]

Heavy metals can be easily absorbed by fishes and vegetables due to their high solubility in the aquatic environments and may accumulate in the human body by means of the food chain. So these toxic heavy metals should be removed from the wastewater to protect the people and the environment. In recent years, a variety of techniques are used for heavy metals removal from water and wastewater which include ion-exchange, adsorption, chemical precipitation, membrane filtration, flocculation, coagulation, flotation and electrochemical methods [3].

Electro-coagulation is an electrochemical approach, which uses an electrical current to remove metals from solution. Electro-coagulation system is also effective in removing suspended solids, dissolved metals, tannins and dyes. The contaminants presents in wastewater are maintained in solution by electrical charges. When these ions and other charged particles are neutralized with ions of opposite electrical charges provided by electrocoagulation system, they become destabilized and precipitate in a stable form. Electrochemical methods are simple, fast, inexpensive, easily operable and eco-friendly in nature. Besides, purified water is potable, clear, colorless and odorless with low sludge production. There is no chance of secondary contamination of water in these techniques.

Electrocoagulation process (EC) has been successfully applied to remove soluble ionic species from solutions and heavy metals by various investigators [11, 12]. The EC process is based on the continuous in situ production of a coagulant in the contaminated water. It had been shown that EC is able to eliminate a variety of pollutants from wastewaters, as for example metals and arsenic [3] strontium and cesium[13], phosphate [14], sulfide, sulfate and sulfite [15], boron [16], fluoride [17], nitrate [18], chromium [19–22], cadmium [23], zinc [24], nickel [25, 26], mercury [27], cobalt [28], clay minerals [29], as well as oil [30], chemical oxygen demand [31], color [32] and organic substances [33].

The most widely used method for the treatment of metal polluted wastewater is precipitation with NaOH and coagulation with FeSO_4_ or Al_2_(SO_4_)_3_ with subsequent time-consuming sedimentation [34]. Other methods include adsorption, ion exchange and reverse osmosis [34]. Although precipitation is shown to be quite efficient in treating industrial effluents, the chemical coagulation may induce secondary pollution caused by added chemical substances [34]. These disadvantages encouraged many studies on the use of electrocoagulation for the treatment of several industrial effluents [34]. This technique does not require supplementary addition of chemicals, reduces the volume of produced sludge [33] and first economic studies indicate also a financial advantage compared to the conventional methods [35].

EC process has the potential to extensively eliminate the disadvantages of the classical treatment techniques to achieve a sustainable and economic treatment of polluted wastewater [33, 36]. Since the turn of the 19th century, EC has been applied for wastewater treatment [37] and many studies attended to optimize the process for specific problems. Typically, empirical studies were done [34, 38]. These studies show the successful treatment of the wastewaters, however, they provide little insight into fundamental chemical and physical mechanisms [39]. Therefore, the mechanisms involved are yet not clearly understood [39]. But exactly these physicochemical mechanisms have to be understood to optimize and control the process, to allow modeling of the method and to improve the design of the system. The main objectives of the present work were to gain insight into some fundamental mechanisms and possible interactions influencing the removal process of heavy metals by electrocoagulation.

Table 2 shows the removal efficiency of heavy metals by various treatment technologies. In addition, removal of some of metals and other pollutants by EC process are presented in Table 3.

**Table 2:** Comparison of various treatment technologies for removal of heavy metals from aqueous environments

Treatment method	Metal	pH of solution	Initial concentration (mg/l)	Efficiency (%)	References
Reverse osmosis	Ni^2+^	3	26	98	[63]
		7	26	99	[63]
	Cu^2+^	3	17	98	[63]
		7	17	99	[63]
	Cr	3	167	95	[63]
		7	167	99	[63]
Ultrafiltration	Ni^2+^	7	50	99	[64]
		7	100	99	[64]
	Cu^2+^	7	50	98	[64]
		7	100	97	[64]
	Cr	7	50	93	[64]
		7	100	76	[64]
	Ni^2+^	-	25	100	[65]
Nanofiltration	Cu^2+^	-	200	96	[66]
Electrocoagulation	Ni^2+^	3	394	98	[67]
		7	394	99	[67]
	Cu^2+^	3	45	100	[67]
		7	45	100	[67]
	Cr	3	44.5	100	[67]
		7	44.5	100	[67]
	Ni^2+^, Zn^2+^	6	248, 270, 282; 217, 232, 236	100	[68]
Chemical precipitation	Cu^2+^, Zn^2+^, Cr^3+^, Pb^2+^	7- 11	100 mg/L	99.3-99.6	[69]
	Cu^2+^, Zn^2+^, Pb^2+^	3	0.01, 1.34, 2.3 mM	100, >94, >92	[70]
Adsorption	Pb^2+^	4	2072	-	[71]
	Pb^2+^	4	1036	55	[72]
	Cd^2+^, Cr^6+^	6	2	Cd^2+^ = 55, Cr^6+^ = 60	[22]

**Table 3:** Removal of heavy metals and other pollutants by EC process

References	Metals or other compounds	Concentration (mg/L)	Anode–cathode	Removal efficiency (%)
[55]	Cr^3+^, Cr^6+^	887.2, 1495.2	Fe-Fe	100
[67]	Cu^2+^, Cr, Ni^2+^	45, 44.5, 394	Al-Fe	100
[49]	Cd^2+^	20	Al-Al	AC: 97.5, DC: 96.2
[18]	NO_3_^−^	150	Fe-Fe, Al-Al	90, 89.7
[23]	Pb^2+^, Zn^2+^, Cd^2+^	170, 50, 1.5	Al-SS	95, 68, 66
[50]	As	150	Al-Al, Fe-Fe	93.5, 94
[26]	TOC, Ni^2+^, Zn^2+^	173, 248, 232	SS _304_-SS _304_	66, 90, 100
[73]	Humic acid	20	Fe-Fe	92.69

Nomenclature: *Cr* chromium, *Ni* nickel, *Cu* copper, *As* arsenic, *Zn* zinc, *pb* lead, *Cd* Cadmium, *Co* cobalt, *Fe* iron, *Al* aluminum, *St* steel, *SS* stainless steel

### Description of electrocoagulation process

Electrocoagulation (EC) is a simple and efficient method and has been used for the treatment of many types of wastewaters such as electroplating wastewater [34], laundry wastewater [40], restaurant wastewater [38] and poultry slaughterhouse wastewater [41]. EC has been successfully used for the removal of pollutants from different industrial wastewaters (Table 4). Many studies have been reported in the literature [20, 21, 24, 42].

**Table 4:** Application of electrocoagulation process for treatment of different types of wastewater

References	Type of wastewater	Current density or current	Time (min)	pH	Anode–cathode	COD removal (%)
[47]	Olive oil mill wastewater	39.06, 78.1 and 117.18 A/m^2^	60	5.2	Ti-Fe	96.14
[74]	Real dairy wastewater	5A	60	7.24	Al-Al	98.84
[75]	Slaughterhouse wastewater	5A	15	7	Al-Al	99
[76]	Carwash wastewater	5 A	15	7.65 ± 0.02	Al-Al	COD = 96.8, BOD_5_ = 94,TSS = 98.4, MBAS = 98.6
[77]	Textile wastewater	5 A	60	7	Al-Al	98.28
[44]	Textile wastewater	-	3	10.6	Fe-Fe	84
[34]	Olive mill effluents	75 mA/cm^2^	25	4-6	Al-Al	76
[37]	Industrial effluents	0.01 A/m^2^	30	10.8	SS-SS	95

Nomenclature: *MS* mild steel, *SS* Stainless steel, *St* steel, *Ti* titanium, *Fe* iron, *Pt* platinum, *Cu* copper

EC in combination with other treatment processes is a safe and effective way for the removal of pollutants. EC is an efficient technique because adsorption of hydroxide on mineral surfaces are 100 times greater on in ‘situ’ rather than on pre-precipitated hydroxides when metal hydroxides are used as coagulant [43]. Since the flocs formed by EC are relatively large which contain less bound water and are more stable, therefore, they can be easily removed by filtration. It is cost effective and easily Performance. EC needs simple equipment’s and can be designed for any capacity of effluent treatment plant. Since no chemical addition is required in this process, it reduces the possibility of generation of secondary pollutants. It needs low current and therefore, can be operated by green processes, such as, solar, windmills and fuel cells [44]. It is an environment friendly technique since the electron is the main reagent and does not require addition of the reagents/chemicals. This will minimize the sludge generation to a great extent and eventually eliminate some of the harmful chemicals used as coagulants in the conventional effluent treatment methods. EC process can effectively destabilize small colloidal particles and generates lower quantity of sludge compared to other processes. The advantages of EC as compared to chemical coagulation are as follows:

1. EC requires simple equipment and is easy to operate with sufficient operational latitude to handle most problems encountered on running. Wastewater treated by EC gives pleasant/edible palatable, clear, colorless and odorless water.
2. Sludge formed by EC tends to be readily settable and easy to de-water, because its main elements/components are metallic oxides/hydroxides. Above all, it is a low sludge producing technique.
3. Flocs formed by EC are similar to chemical flocs, except that EC flocs tends to be much larger, contains less bound water, is acid-resistant and more stable and therefore, can be separated faster by filtration.
4. EC produces effluent with less total dissolved solids (TDS) content as compared with chemical treatments. If this water is reused, the low TDS level contributes to a lower water recovery cost.
5. The EC process has the advantage of removing the smallest colloidal particles, because the applied electric field sets them in faster motion, thereby facilitating the coagulation. The EC process avoids uses of chemicals and so there is no problem of neutralizing excess chemicals and no possibility of secondary pollution caused by chemical substances added at high concentration as when chemical coagulation of wastewater is used.
6. The gas bubbles produced during electrolysis can carry the pollutant to the top of the solution where it can be more easily concentrated, collected and removed. The electrolytic processes in the EC cell are controlled electrically with no moving parts, thus requiring less maintenance.

The EC technique can be conveniently used in rural areas where electricity is not available, since a solar panel attached to the unit may be sufficient to carry out the process. Potentially recoverable metals and reuse of treated effluent are other advantages of EC. EC is an alternative to chemical precipitation for the removal of dissolved and suspended metals in aqueous solutions (see Chemical Precipitation Technology Overview). The quantity of sludge produced is lower. The floc generated is larger and heavier and settles out better than in conventional chemical precipitation processes. Since a large thickener is not required, capital costs can also be lower. The effluent generated by EC contains no added chemicals and is often of better quality, containing TDS and less colloidal particulates. Reduction of TDS has been reported at 27 %-60 %, and reduction of total suspended solids can be as great as 95 %-99 % [45].

Although EC requires energy input, it requires only low currents and can be operated using green technologies such as solar or wind power. Some of the limitations of the electrochemical coagulation are as follows [43, 46]:

1. The sacrificial anodes need to be replaced periodically.
2. EC requires minimum solution conductivity depending on reactor design, limiting its use with effluent containing low dissolved solids.
3. In case of the removal of organic compounds, from effluent containing chlorides there is a possibility of formation of toxic chlorinated organic compounds.
4. An impermeable oxide film may be formed on the cathode which may provide resistance to the flow of electric current. However, change of polarity and periodical cleaning of the electrodes may reduce this interference.
5. The high cost of electricity can result in an increase in operational cost of EC process [43].

Electrocoagulation process involves the generation of coagulants in situ by dissolving electrically either aluminum or iron ions from aluminum or iron electrodes, respectively. In this process, the metal ions generation takes place at the anode and hydrogen gas is released from the cathode. The hydrogen gas bubbles carry the pollutant to the top of the solution where it can be more easily concentrated, collected and removed. Various reactions take place in the electrocoagulation process, where aluminum is used as the electrode:

At the anode:

1 $$ \mathrm{A}\mathrm{l}\to {{\mathrm{Al}}^{3+}}_{\left(\mathrm{a}\mathrm{q}\right)}+\kern0.5em 3\mathrm{e} $$

At the cathode:

2 $$ 3{\mathrm{H}}_2\mathrm{O} + 3\mathrm{e}\ \to 3/2{\mathrm{H}}_2 + 3{\mathrm{OH}}^{-} $$

The cathode may also be chemically attacked by OH^−^ ions generated during H_2_ evolution at high pH:

3 $$ 2\mathrm{A}\mathrm{l} + 6{\mathrm{H}}_2\mathrm{O} + 2{\mathrm{OH}}^{-}\to 2\mathrm{A}\mathrm{l}{{\left(\mathrm{O}\mathrm{H}\right)}_4}^{-} + 3{\mathrm{H}}_2 $$

Al^3+^_(aq)_ and OH^−^ ions generated by electrode reactions (1) and (2) react to form various monomeric species such as Al(OH)^2+^, Al(OH)_2_^+^, Al_2_(OH)_2_^4 +^, Al(OH)_4_^−^, and polymeric species such as Al_6_(OH)_15_^3 +^, Al_7_(OH)_17_^4 +^, Al_8_(OH)_20_^4 +^, Al_13_O_4_(OH)_24_^7 +^, Al_13_(OH)_34_^5 +^, which transform finally into Al(OH)_3_ according to complex precipitation kinetics [43].

Freshly formed amorphous Al(OH)_3_ “sweep flocs” have large surface areas which are beneficial for a rapid adsorption of soluble organic compounds and trapping of colloidal particles. These flocs polymerize as:

4 $$ n\mathrm{A}\mathrm{l}{\left(\mathrm{O}\mathrm{H}\right)}_3\to {\mathrm{Al}}_n{\left(\mathrm{O}\mathrm{H}\right)}_{3n} $$

and they are easily removed from aqueous environment by sedimentation and by H_2_ flotation. Secondary anodic reactions occur also during electrocoagulation process for example, in neutral and acidic chloride solutions, native and free chlorine and hypochlorite are formed which are strong oxidants. On the other hand, the aluminum hydroxide flocs normally act as adsorbents and/or traps for pollutants. Therefore, they would eliminate them from the solution [43].

In addition the main reactions occurring at the iron electrodes are:

5 $$ \mathrm{F}\mathrm{e}\ \left(\mathrm{s}\right)\leftrightarrow\ {{\mathrm{Fe}}^{+3}}_{\mathrm{aq}} + 3{\mathrm{e}}^{-}\left(\mathrm{anode}\right) $$

6 $$ 3{\mathrm{H}}_2\mathrm{O} + 3{\mathrm{e}}^{-}\leftrightarrow 3/{{2\ \mathrm{H}}_2}_{\mathrm{g}} + {{3\mathrm{O}\mathrm{H}}^{-}}_{\mathrm{aq}}\left(\mathrm{cathode}\right) $$

In addition, Fe^3+^ and OH^−^ ions generated at electrode surfaces react in the bulk wastewater to form ferric hydroxide:

7 $$ {{\mathrm{Fe}}^{+3}}_{\mathrm{aq}} + {{3\mathrm{O}\mathrm{H}}^{-}}_{\mathrm{aq}}\leftrightarrow \mathrm{F}\mathrm{e}{\left(\mathrm{O}\mathrm{H}\right)}_3 $$

The suspended aluminum or iron hydroxides can remove pollutants from the solution by sorption, co-precipitation or electrostatic attraction, followed by coagulation [43].

For a particular electrical current flow in an electrolytic cell, the mass of aluminum or iron theoretically dissolved from the sacrificial anode is quantified by Faraday’s law [43]:

8 $$ w=\left[\frac{ItM}{ZF}\right] $$

where “w” is the amount of anode material dissolved (g), I the current (A), the electrolysis time (t), M the specific molecular weight of electrode (g/mol), Z the number of electrons involved in the reaction and F is the Faraday’s constant (96485.34 C/mol). The mass of evolved hydrogen and formed hydroxyl ions can be calculated correspondingly. The amount of coagulant dosed into the solution can be increased by increasing the current and the reaction time. But increasing the current density leads to a decreased current efficiency. Some influencing factors of the EC process are current density (or applied voltage), conductivity and pH of solution, mode of operation, electrolysis time, electrode material and distance between the electrodes [43].

### Batch and continuous mode of operation

**Fig. 1:**
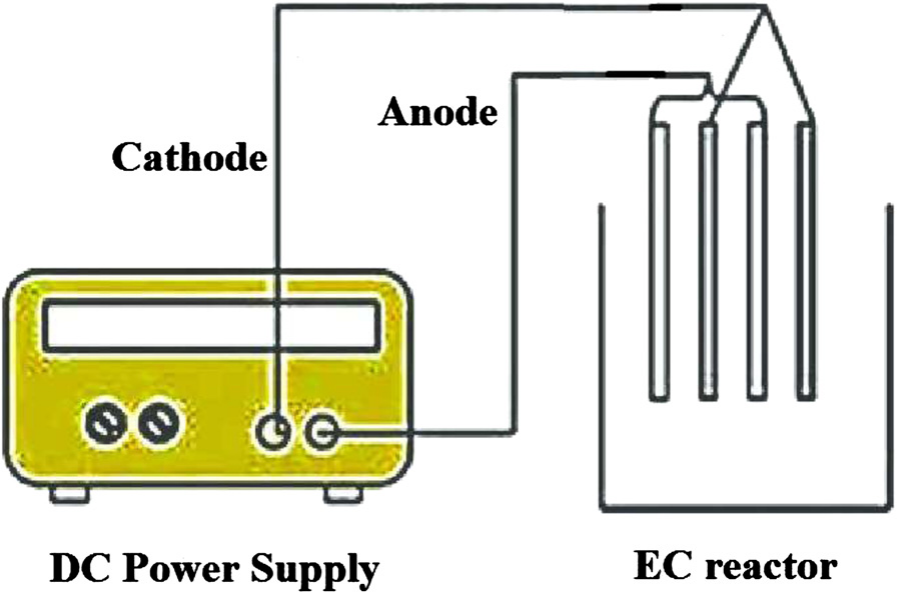
Batch electrochemical reactor

**Fig. 2:**
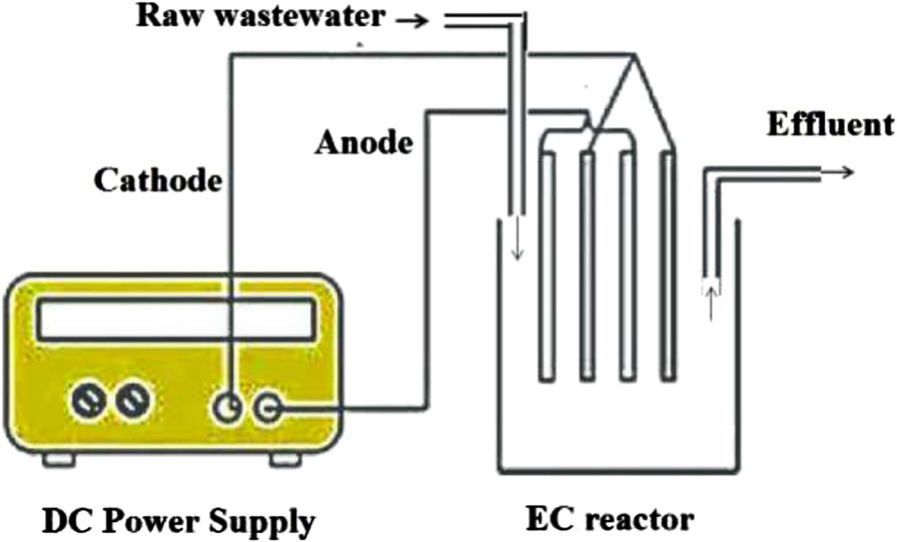
Continues flow electrochemical reactor

It can be noticed from the literature that EC has been studied for the removal of a wide range of pollutants using batch and continuous mode of operation. (Diagram of batch and continues flow electrochemical reactor is shown in Figs. 1 and 2). A continuous system operates under steady state conditions, specially a fixed pollutant concentration and effluent flow rate. Comparably, a batch reactor’s dynamic nature enables to study the range of operating conditions and is more suited for research work [45]. Continuous systems are better suited to industrial processes for large effluent volumes whereas the batch reactors are suited to laboratory and pilot plant scale applications. The continuous mode of operation is preferred due to its better control than the batch mode of operation.

Batch mode of EC reactors exhibits time dependent behavior as the coagulant is continuously generated in the reactor with the dissolution of anode. The anode material is hydrolyzed, and is capable of aggregating the pollutants. As a result, the concentration of the pollutant, coagulant, and pH keeps on changing with respect to time. A batch reactor has neither inflow nor outflow of effluent during the electrolysis time [45].

### Effect of various operating parameters on pollutants removal

The efficiency of the EC process depends on many operational parameters such as conductivity of the solution, arrangement of electrode, electrode shape, type of power supply, pH of the solution, current density, distance between the electrodes, agitation speed, electrolysis time, initial pollutant concentration, retention time and passivation of the electrode.

### Solution conductivity and type of power supply

Conductivity of the solution is very important parameter in electrolysis process as the removal efficiency of the pollutant and operating cost are directly related to the solution conductivity [45]. The conductivity of an electrolyte solution is a key property. In an electrochemical process, the conductivity determines the cell resistance while the properties of solvent and electrolyte determine their interaction with the electrically active species and thereby influence the electrode reactions [47].

The solution must have some minimum conductivity for the flow of the electric current. The conductivity of the low-conductivity wastewater is adjusted by adding sufficient amount of salts such as sodium chloride or sodium sulphate. There is an increase in the current density with an increase in the conductivity of the solution at constant cell voltage or reduction in the cell voltage at constant current density [48]. The energy consumption is decreased with high performance/approach solution. The energy consumption is decreased with high conductivity solution. In the EC process, there is an in situ generation of metal hydroxide ions by electrolytic oxidation of the sacrificial anode. These metal hydroxide ions act as coagulant and remove the pollutants from the solution by sedimentation. Majority of the studies reported in the literature have used direct current (DC) in the EC process. The use of DC leads to the corrosion formation on the anode due to oxidation. An oxidation layer also form on the cathode reducing the flow of current between the cathode and the anode and thereby lowering the pollutant removal efficiency [49]. These limitations of the DC electrocoagulation process have been decreased to some extent by the addition of parallel plate sacrificial electrodes in the cell configuration. Nevertheless, many have preferred the use of alternating current electrocoagulation (ACE) technology. It is believed that the ac cyclic energization retards the normal mechanisms of electrode attack that are experienced in DC electrocoagulation system, and consequently, ensure reasonable electrode life. In addition to that, since the AC electric fields in an ACE separator do not cause electrophoretic transport of the charged particles due to the frequent change of polarity, it can induce dipole–dipole interactions in a system containing non spherical charged species. Consequently, the AC electric fields may also disrupt the stability of balanced dipolar structures existing in such a system. This is, however, not possible in a DC electrocoagulation separator using DC electric fields [46].

### Arrangement of electrodes

**Fig. 3:**
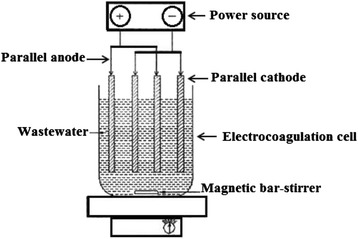
Bench-scale EC reactor with monopolar electrodes in parallel connection (46)

**Fig. 4:**
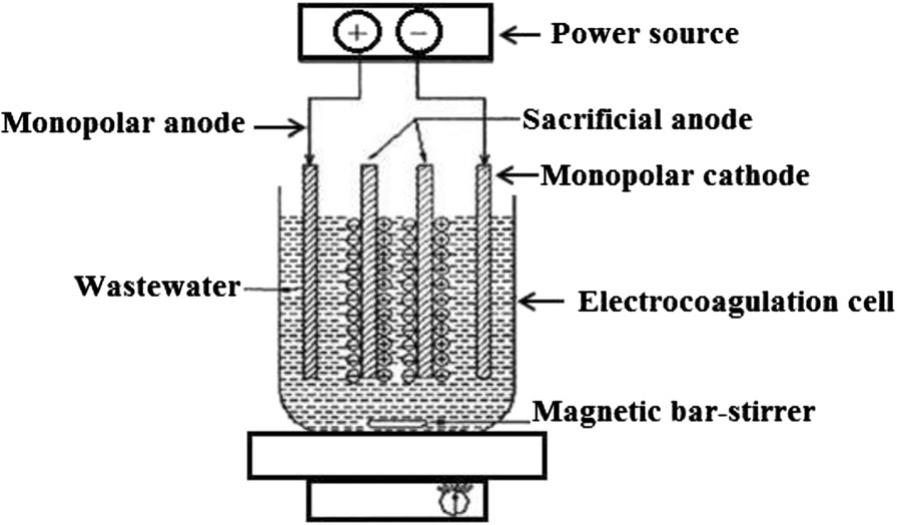
Bench-scale EC reactor with bipolar electrodes in parallel connection (46)

The electrode material and the connection mode of the electrodes play a significant role in the cost analysis of the EC process. Kobya et al. [50] studied the treatment of textile wastewater and compared the performances of various electrode connection modes as a function of wastewater pH, current density and operating time. They studied three different modes of electrode connection and areas follow: Monopolar electrodes in parallel connections (MP-P): The anodes and cathodes are connected in parallel due to which the current is divided between all the electrodes to the resistance of individual cells. The parallel connection needs a lower potential difference compared with serial connections [50]. Monopolar electrodes in serial connections (MP-S): In the monopolar configuration, each pair of sacrificial electrodes is internally connected with each other. The addition of the cell voltages leads to a higher potential difference for a given current. Bipolar electrode in serial connections (BP-S): In this connection mode, the outer electrodes are connected to the power supply and there is the no electrical connection between the inner electrodes [50]. Schematic diagram of EC reactor with monopolar and bipolar electrode connections is shown in Figs. 3 and 4.

The shape of the electrodes affects the pollutant removal efficiency in the EC process. It is expected that the punched holes type electrodes will result in higher removal efficiency compared to the plane electrodes. Very few studies have been reported in the literature [51] describing the effect of electrode shape on the performance of the electrostatic precipitator. Kuroda et al. [51] performed experiments using metallic electrodes with/without punched holes as a barrier discharge electrode to study the effect of electrode shape of reactor on the collector efficiency in electrostatic precipitator. They have reported higher discharge current for the electrode with punched holes than for plane electrode resulting in higher collection efficiency with punched electrode compared with plane electrode. The electric field intensity at the edge of punched holes type electrodes is higher (1.2 times) than at plane type electrode resulting in an increase in the discharge current at punched type electrode. More studies are needed to establish the effect of the electrode shape (punched hole diameter and pitch of the holes) on the EC process [51].

### Current density

Current density is very important parameter in EC as it determines the coagulant dosage rate, bubble production rate, size and growth of the flocs, which can affect the efficiency of the EC. With an increase in the current density, the anode dissolution rate increases. This leads to an increase in the number of metal hydroxide flocs resulting in the increase in pollutant removal efficiency. An increase in current density above the optimum current density does not result in an increase in the pollutant removal efficiency as sufficient numbers of metal hydroxide flocs are available for the sedimentation of the pollutant [52, 53]. Effect of current density or current on removal efficiency of EC process is shown in Table 5.

**Table 5:** Effect of cell voltage (V), electrode material, electrode connection mode, current or current density, flow rate and pH on removal efficiency of heavy metals in EC process

References	Heavy metals	Current density	Cell voltage (V)	Flow rates	Optimum pH	Electrode materials	Removal efficiency (%)
[78]	Cr^6+^	8.33 A/m^2^	2	1.2 m^3^/h	7-8.5	Fe-Fe A_M_	70-85
[34]	Cu^2+^, Zn^2+^, Cr^6+^	4.8A/dm^2^	-	10 ml/min	4	Al-Al	99, 99, 83
[19]	Cr^6+^	30 A/m^2^	-	50 ml/min	5–8	Fe-Fe	80-97
[21]	Cr^6+^	5A	40	-	10	Al-Al	99
[79]	Cr^3+^	A_M_ = 10.84, Bi = 32.52 mA/cm^2^	-	40 ml/min	A_M_ = 5.5, Bi = 6	MS-MS	A_M_ = 90.6, Bi = 71.4
[20]	Cr^6+^	5A	20-40	-	3	Al-Fe	99.9
[80]	Cr^6+^	0.05, 1 A	30	-	5	Al-Al	100
[81]	Cr^6+^	35.7 mA/ cm^2^	10–24	22.5 ml/min	5	Al–Al	90.4
[82]	Cr^6+^	55.5 mA/cm^2^	60	12 ml/min	7	Fe-Fe, Pt Ti (platinized titanium)/Fe, Al/Al and Pt Ti/Al	65.3
[12]	Cr^6+^	5A	40	-	3	Fe-Fe	98
[83]	Cr^6+^	153 A/m^2^	15-25	-	5	Fe-Al	99
[84]	Cr^6+^	2-25 mA/cm^2^	80	-	5.68	Fe-Fe	99
[55]	Cr^3+^, Cr^6+^	50 mA/cm^2^	-	-	4	Fe-Fe	100
[85]	Fe, Ni^2+^, Cu^2+^, Zn^2+^, Pb^2+^, Cd^2+^	11.55 mA/cm^2^	0-30	-	7.6	Al-Al	SS = 86.5, turbidity = 81.56, BOD_5_ = 83, COD = 68, color > 92.5
[86]	Cd^2+^, Cu^2+^	5 A	30	20 L/h	0.64	Ss-ti	Cd^2+^ = 73.8,Cu^2+^ = 98.8
[87]	Cd^2+^	2.2, 3.5 mA/cm^2^	6	-	11	Al-Fe	>99.5
[88]	Cd^2+^	0.2 A/dm^2^	AC:270, DC: 25	-	7	Zn-Zn	AC: 97.8, DC: 96.9
[49]	Cd^2+^	0.2 A/dm^2^	DC = 25,AC = 270	-	7	Al-Al	AC: 97.5, DC: 96.2
[89]	Cd^2+^	0.04 A/m^2^	70	5 ml/min	8.9	Al-Al	98.2
[90]	Zn phosphate	60.0 A/m^2^	30	400 mL/min	Al-Al = 5, Fe-Fe = 3	Al-Fe	max 97.8
[24]	Zn^2+^	15 mA cm^2^	60	-	6	Al-Fe	>99
[42]	Zn^2+^, Cu^2+^	5A	40	-	7	Fe-Fe	99.99
[58]	COD, Zn^2+^	COD = 0.90, Zn^2+^ = 0.45-1.8 A/dm^2^	-	-	COD = 3, ZN^2+^ = 10	Fe-graphite	COD = 88, 99.3 Zn^2+^ = 99
[91]	Ni^2+^, Cu^2+^	0.3 A	29	-	5	RO-Ti-Ss	Ni^2+^ = 82, Cu^2+^ = 99
[12]	Zn^2+^, Cu^2+^	5 A	40	-	7	Al-Al	Zn^2+^ = 99.6, Cu^2+^ = 99.9
[67]	Cu^2+^, Cr, Ni^2+^	10 mA/cm^2^	30	-	3	Fe-Al	100
[17]	Ni^2+^	5 A	20	-	10	Fe-Fe	99.99
[50]	As	Al = 2.5 A/m^2^, Fe =7.5 A/m^2^	Al electrode = 0.8-1.6, Fe electrode =1.5-2.3	Fe electrode = 60 ml/min, Al electrode = 50 ml/min	Fe = 6.5, Al =7	Al-Al, Fe-Fe	Fe = 93.5, Al = 95.7
[92]	As, Nitrite	-	NO_3_ = 25, As^5+^ = 20	2 L/h	9.5	MS-MS	NO_3_ = 84, As^5+^ = 75
[93]	As	8.86 mA/cm^2^	17	7 L/h	5 ± 0.2	AL-AL	89
[11]	Oil, grease, heavy metals	0.6 A/cm^2^	40	1 L/min	2-4	Al-Cs	Zn^2+^ = 99, Cu^2+^, Ni^2+^ = 70, Oil and grease = 99.9, Turbidity = 99.7
[28]	Co	6.25 mA·/cm^2^	30	-	8	Al-Al	99
[94]	Heavy metals	4 mA/cm^2^	30	600 mL/min	9.56	Cs-Cs	Cr^3+^ = Cu^2+^ = 100, Ni^2+^ = 99
[16]	Boron	12.5 mA/cm^2^	30	-	6.3	Al-Al	99.7
[95]	Ba , Zn^2+^, Pb^2+^	350 A/m^2^	30	-	10	Ss-Ss	97
[96]	Cd^2+^	3.68 mA/ cm^2^	-	-	Bipolar configuration = 10.90, monopolar configuration = 9.03	AL- AL	100
[97]	Ni^2+^	7.5 A/m^2^	-	6 ml/min	6	AL- AL, Fe- Fe	100
[98]	Cr^6+^	0.55 A	20	-	1	Fe-Fe	100
[99]	Zn^2+,^ Cu^2+^, Ni^2+^, Ag^+^, Cr_2_O_7_^2−^	33 A/m^2^	30	-	9	Al–Al	>50
[100]	Cr^6+^	50-200 A/m^2^	-	2.5 cm^3^/ s	7.5	Fe-Fe, Al-Al	40

Nomenclature: *MS* mild steel, *SS* stainless steel, *St* steel, *Ti* titanium, *Fe* iron, *Pt* platinum, *Cu* copper, *CS* carbon steel electrodes, *RuO* ruthenium oxide, *A*_*M*_ Monopolar, *B*_*i*_ Bipolar

### Distance between the electrodes

Inter-electrode spacing is a vital parameter in the reactor design for the removal of pollutant from effluent. The inter electrode-spacing and effective surface area of electrodes are important variable when an operational costs optimization of a reactor is needed [52]. To decrease the energy consumption (at constant current density) in the treatment of effluent with a relatively high conductivity, larger spacing should be used between electrodes. For effluent with low conductivity, energy consumption can be minimized by decreasing the spacing between the electrodes [53].

The inter electrode distance plays a significant role in the EC as the electrostatic field depends on the distance between the anode and the cathode. The maximum pollutant removal efficiency is obtained by maintaining an optimum distance between the electrodes. At the minimum inter electrode distance; the pollutant removal efficiency is low. This is due to the fact that the generated metal hydroxides which act as the flocs and remove the pollutant by sedimentation get degraded by collision with each other due to high electrostatic attraction [54]. The pollutant removal efficiency increases with an increase in the inter electrode distance from the minimum till the optimum distance between the electrodes. This is due to the fact that by further increasing the distance between the electrodes, there is a decrease in the electrostatic effects resulting in a slower movement of the generated ions. It provides more time for the generated metal hydroxide to agglomerate to form the flocs resulting in an increase in the removal efficiency of the pollutant in the solution. On further increasing the electrode distance more than the optimum electrode distance, there is a reduction in the pollutant removal efficiency. This is due to the fact that the travel time of the ions increases with an increase in the distance between the electrodes. This leads to a decrease in the electrostatic attraction resulting in the less formation of flocs needed to coagulate the pollutant [54]. The pollutant removal efficiency is low at the minimum inter electrode distance. Effect of distance between the electrodes and also type of reactor (batch or continuous) on removal efficiency of EC process are presented in Table 6.

**Table 6:** Effect of inter electrodes distance, conductivity of solutions, energy consumption and electrolysis time on heavy metals removal efficiency in EC process

References	Heavy metal	Reactor	Electrolysis time	Inter electrode distance	Conductivity (mS/cm)	Energy consumption	Efficiency (%)
[78]	Cr^6+^	Continuous	10-12 min	-	-	-	70-85
[34]	Cu^2+^, Zn^2+^, Cr^6+^	Continuous	20 min	5 mm	-	-	99, 99, 83
[19]	Cr^6+^	Continuous	72 min	4 mm	1.5	1 kWh/m^3^	80-97
[21]	Cr^6+^	Batch	20 min	1.5 cm	1.6	1.92-2.29 kwh/m^3^	99
[79]	Cr^3+^	Continuous	20-25 min	22 mm	5.73 , 7.36	0.1KWh/m^3^	A_M_ = 90.6, Bi = 71.4
[20]	Cr^6+^	Batch	20, 60 min	1.5 cm	1.6	2.11 kWh/m^3^	99.9
[80]	Cr^6+^	Batch	45 min	5 mm	20	9.0 kWh/m^3^	100
[81]	Cr^6+^	Continuous	24 min	15 mm	2	137.2 KWh/m^3^	90.4
[82]	Cr^6+^	Continuous	75 min	4 cm	2.41, 1.70	-	65.3
[12]	Cr^6+^	Batch	60 min	1.5 cm	1.6	35.06 kwh/g	98
[83]	Cr^6+^	Batch	25 min	1.5 cm	0.59- 3.4	16.3 kWh//m^3^	99
[84]	Cr^6+^	Batch	5-10 min	0.3 cm	365	38 kWh/m^3^	99
[55]	Cr^3+^, Cr^6+^	Batch	15 min	0.5 cm	-	-	100
[85]	Fe, Ni^2+^, Cu^2+^, Zn^2+^, Pb^2+^, Cd^2+^	Batch	10 min	1 cm	2.1	-	SS = 86.5, Turbidity = 81.56, BOD_5_ = 83, COD = 68, Color > 92.5
[19]	Cd^2+^	Batch	20 min	1.5 cm	-	9.37 kwh/kg	>99
[86]	Cd^2+^, Cu^2+^	Continuous	120 min	1.5 cm	-	10.99 kWh/kg	Cd^2+^ = 73.8,Cu^2+^ = 98.8
[87]	Cd^2+^	Batch	10 min	-	1.05- 5.22	-	>99.5
[88]	Cd^2+^	Batch	30 min	5 mm	-	AC:0.6, DC: 1.2 kWh/m^3^	AC: 97.8, DC: 96.9
[49]	Cd^2+^	Batch	AC: 30, DC: 45 min	5 mm	-	AC:0.4, DC:1 kWh/ kg	AC: 97.5, DC: 96.2
[89]	Cd^2+^	Continuous	200 min	1 cm	1.06	-	98.2
[90]	Zn phosphate	Batch and continuous	15 min = Fe electrode, 25 min = Al electrode	Batch = 11, continuous = 20 mm	Batch = 5.1-5.3, Continuous = 4.8- 4.9	Al electrode =0.18–11.29, Fe electrode = 0.24-8.47 kWh/m^3^	Max 97.8
[24]	Zn^2+^	Batch	10 min	11 mm	3000 μS/cm	3.3 kWh/kg	>99
[42]	Zn^2+^, Cu^2+^	Batch	60 min	1.5 cm	1.6	Zn^2+^ = 22.31, Cu^2+^ = 35.63KWh/g	99.99
[58]	COD, Zn^2+^	Batch	50 min	16 mm	0.49	1.7 kWh/kg	COD = 88, 99.3, Zn^2+^ = 99
[91]	Ni^2+^, Cu^2+^	Batch	60 min	1 cm	634 μS/cm	-	Ni^2+^ = 82, Cu^2+^ = 99
[12]	Zn^2+^, Cu^2+^	Batch	60 min	1.5 cm	1.6	Zn^2+^ = 19.98, Cu^2+^ = 35.06 kWh	Zn^2+^ = Cu^2+^ = 99.9
[67]	Cu^2+^, Cr, Ni^2+^	Batch	20 min	10 mm	2	10.07 kWh/m^3^	100
[17]	Ni^2+^	Batch	20, 40 min	1 cm	1.6	9.37 kWh/kg	99.9
[50]	As	Continuous	Fe electrode =12.5, Al electrode = 15 min	13 mm	1.55	Fe electrode =0.015, Al electrode = 0.032 kWh/m^3^	Fe = 93.5, Al = 95.7
[92]	As^5+^,NO_2_^−^	Continuous	120 min	7 cm	-	-	Nitrite = 84, As^5+^ = 75
[93]	As	Continuous	30 min	1.2 cm	1700 ± 37 μS/ cm	3.03 kWh/m^3^	89
[11]	Oil, grease, heavy metals	Continuous	105 s	-	-	Cu^2+^, Ni^2+^ = 0.166, Zn^2+^ = 0.117, Oil and grease = 0.116, Turbidity = 0.117 kwh/m^3^	Zn^2+^ = 99, Cu^2+^, Ni^2+^ = 70, Oil and grease = 99.9, Turbidity = 99.7
[28]	Co	Batch	15 min	-	6.5	3.3 kwh/m^3^	99
[94]	Heavy metal	Continuous	45 min	15 mm	8.9 ± 0.2	6.25 kWh/m3	Cr^3+^ = Cu^2+^ = 100, Ni^2+^ = 99
[16]	Boron	Batch	89 min	0.5 cm	30,000 mS/cm	2.4 kWh/m3	99.7
[95]	Ba, Zn^2+^, Pb^2+^	Continuous	20 min	10 mm	-	14 kWh/m3	97
[96]	Cd^2+^	Batch	5 min	0.5 cm	1.176 mS/cm	1.6 kW h m 3	100
[97]	Ni^2+^	Continuous	20 min	10 mm	1 mS/cm	-	100
[98]	Cr^6+^	Batch	14 min	0.87 cm	adjusted	0.007 kWh/g	100
[99]	Zn^2+,^ Cu^2+^, Ni^2+^, Ag^+^, Cr_2_O_7_^2−^	Batch	30 min	5 mm	20 mS /cm	-	>50
[100]	Cr^6+^	Continuous	60 min	20 mm	2.4 mS /cm	-	40

### Electrolysis time

The pollutant removal efficiency is also a function of the electrolysis time. The pollutant removal efficiency increases with an increase in the electrolysis time. But beyond the optimum electrolysis time, the pollutant removal efficiency becomes constant and does not increase with an increase in the electrolysis time. The metal hydroxides are formed by the dissolution of the anode. For a fixed current density, the number of generated metal hydroxide increases with an increase in the electrolysis time. For a longer electrolysis time, there is an increase in the generation of flocs resulting in an increase in the pollutant removal efficiency. For an electrolysis time beyond the optimum electrolysis time, the pollutant removal efficiency does not increase as sufficient numbers of flocs are available for the removal of the pollutant [45]. Bazrafshan et al. [20] determined that Cr^6+^ reduction from synthetic chromium solution could be under legal limits as long as treatment was between 20 and 60 minutes. Effect of different electrolysis time on removal efficiency of EC process is shown in Table 6.

### Effect of initial pH on the efficiency of Heavy metal removal

It has been established that pH is an important parameter influencing the performance of the electrochemical process [38]. The maximum pollutant removal efficiency is obtained at an optimum solution pH for a particular pollutant. The precipitation of a pollutant begins at a particular pH. The pollutant removal efficiency decreases by either increasing or decreasing the pH of the solution from the optimum pH. Verma et al. [55] studied the removal of hexavalent chromium from synthetic solution using EC and found that the pH of the solution has a significant effect on the Cr^6+^ removal efficiency. They performed the experiments at different pH of the synthetic solution and obtained the maximum chromium removal efficiency at the pH 4. They further reported that the pH of the synthetic solution after the EC process increased with an increase in the electrolysis time due to the generation of OH in the EC process [55].

The pH changed during batch EF, Its evolution depended on the initial pH. EF process exhibits some buffering capacity because of the balance between the production and the consumption of OH [56].The pH has a significant influence on the coagulant species formed during coagulation processes. It also has influence on the superficial charge of the aluminum hydroxide precipitates (caused by the adsorption of ionic species) [57]. During the time-course of coagulation and EC processes, the pH changes in an opposite way and this affects significantly to the coagulant species formed, and hence to the efficiencies obtained in the removal of pollutants [57].

It cannot be said that any process is better than the other for all wastes. Under the same fluid dynamic conditions, doses of aluminum, pH, the efficiencies obtained by coagulation and EC are very similar. The pH of the waste can be a key parameter in the choice of the coagulation technology [57]. Effect of different initial pH on removal efficiency in EC process is shown in Table 5.

### Cost analysis

Cost analysis plays an important role in industrial wastewater treatment procedure/method as the wastewater treatment technique should be cost attractive. The costs involved in EC include, the cost of energy consumption, cost of the dissolved electrode (electrode consumption) and the cost of addition of any external chemical (for increasing the solution conductivity or varying the pH of the solution).

Electrode consumption can calculate by equation 8 which presented earlier. In addition, electrical energy consumption is a very important economical parameter in the electrocoagulation process and can calculated using the following equation [33]:

9 $$ E=\left[\frac{UIt}{1000\kern0.22em V}\right] $$

where E is the energy consumption (kWh/m^3^), U is the applied voltage (V), I is the current intensity (A), t is the electrocoagulation time (h), and V is the volume of the treated wastewater (m^3^).

The detailed calculation of operating cost for the treatment of fluoride containing drinking water using EC has been reported by Ghosh et al. [58]. Espinoza-Quinones et al., (2009) studied the removal of organic and inorganic pollutants from a wastewater of lather finishing industrial process using EC. They found the EC to be cheaper compared to the conventional method. The operational cost for the EC was found to be US $ 1.7 per cubic meter of the treated tannery effluent as compared to the cost of US $ 3.5 per cubic meter of the treated effluent for conventional methods [59]. Similarly Bayramoglu et al. [60] have been reported that the operating cost of chemical coagulation is 3.2 times as high as that of EC for the treatment of textile wastewater.

## Conclusions

The rapid urbanization and industrialization in the developing countries are creating high levels of water pollution due to harmful industrial effects and sewage discharges. The characteristics of industrial effluents in terms of nature of contaminates, their concentrations, treatment technique and required disposal method vary significantly depending on the type of industry. Further, the choice of an effluent treatment technique is governed by various parameters such as contaminants, their concentration, volume to be treated and toxicity to microbes. Electrocoagulation is a treatment process that is capable of being an effective treatment process as conventional methods such as chemical coagulation. Having observed trends over the last years, it has been noted that electrocoagulation is capable of having high removal efficiencies of color, chemical oxygen demand (COD), biochemical oxygen demand (BOD_5_) and achieving a more efficient treatment processes quicker than traditional coagulation and inexpensive than other methods of treatment such as ultraviolet (UV) and ozone. Unlike biological treatment which requires specific conditions, therefore limiting the ability to treat many wastewaters with high toxicity, xenobiotic compounds, and pH, electrocoagulation can be used to treat multifaceted wastewaters, including industrial, agricultural, and domestic. Continual research using this technology will not only improve new modeling techniques can be used to predict many factors and develop equations that will predict the effectiveness of treatment.

Electrocoagulation is an attractive method for the treatment of various kinds of wastewater, by virtue of various benefits including environmental capability, versatility, energy efficiency, safety, selectivity and cost effectiveness. The process is characterized by simple equipment, easy operation, less operating time and decreased amount of sludge which sediments rapidly and retain less water. However, further studies needs to be performed to study the effect of shape and geometry of the electrodes (punched hole and pitch of the holes) to possibly improve the pollutant removal efficiency. Efforts should be made to study the phenomena of electrode passivation to reduce the operating cost of the EC process. Most of the studies reported in the literature have been carried out at the laboratory scale using synthetic solutions. Efforts should be made to perform EC experiments at pilot plant scale using real industrial effluent to explore the possibility of using EC for treatment of real industrial effluents.
